# RETRACTION: Cosentyx Alleviates Psoriasis‐Induced Podocyte Injury by Inhibiting the TLR/NF‐κB Signaling Pathway

**DOI:** 10.1111/srt.70213

**Published:** 2025-07-22

**RETRACTION**: W. Aixue, W. Feng, Z. Huanhuan, M. Xixing, and L. Yanling, “Cosentyx Alleviates Psoriasis‐Induced Podocyte Injury by Inhibiting the TLR/NF‐κB Signaling Pathway,” *Skin Research and Technology* 30, no. 2 (2024): e13562, https://doi.org/10.1111/srt.13562.

The above article, published online on 26 January 2024 in Wiley Online Library (wileyonlinelibrary.com), has been retracted by agreement between *Skin Research and Technology*; and John Wiley & Sons Ltd. The article was accepted for publication following an insufficient peer review process that does not meet the standards of the journal's peer review policy. Further investigation by the publisher uncovered concerns regarding compliance with ethical standards for animal experimentation. Accordingly, the article has been retracted. The authors have been informed of this decision.

